# β‐Lactam allergy testing and delabeling—Experiences and lessons from Singapore

**DOI:** 10.1002/iid3.318

**Published:** 2020-06-07

**Authors:** Yee Kiat Heng, Yasmin Chia Chia Liew, Yan Ling Kong, Yen Loo Lim

**Affiliations:** ^1^ National Skin Centre Singapore Singapore

**Keywords:** allergy, anaphylaxis, β‐lactam, drug hypersensitivity, drug provocation test, penicillin, skin‐testing

## Abstract

**Background:**

β‐Lactam allergy is over‐reported and this leads to greater healthcare costs. Allergy testing has inherent risks, yet patients who test negative may continue avoiding β‐lactams.

**Objective:**

To evaluate the safety and diagnostic value of β‐lactams allergy testing locally and usage of antibiotics following negative testing.

**Methods:**

We performed a retrospective medical record review and follow‐up survey of patients who underwent β‐lactam testing between 2010 and 2016 at the National Skin Centre, Singapore.

**Results:**

We reviewed the records of 166 patients, with a total of 173 β‐lactam allergy labels. Eighty (46.2%) labels were to penicillin, 75 (43.1%) to amoxicillin/amoxicillin‐clavulanic acid, 11 (6.4%) to cephalexin, and 5 (2.9%) to others. Skin tests were performed in 142 patients and drug provocation tests (DPTs) in 141 patients. Eleven (6.6%) patients defaulted DPTs after skin testing. Out of 166 patients, 22 (13.3%) patients were proven allergic by either skin tests (16) or DPTs (6). Patients who tested positive had nonsevere reactions.

Out of 155 patients who were conclusively evaluated, 133 (85.8%) were not allergic. Of these patients, 30 (22.6%) used the tested β‐lactam subsequently, with one reporting a mild reaction. Fifty‐one (38.3%) patients were uncontactable or uncertain if they consumed a β‐lactam since testing negative. Fifty‐two (39.1%) patients had no re‐exposure (35 had no indication, 17 were fearful of reactions).

**Conclusion:**

Drug allergy testing was safe and removed inappropriate labels.

**Clinical Implication:**

Allergy testing is efficacious, but fears of subsequent rechallenge should be addressed to maximize the effectiveness of allergy delabeling.

## Graphical abstract

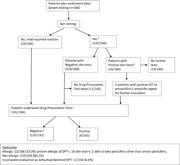

## 1 INTRODUCTION

β‐Lactams are the most commonly used antibiotics in the world.^1^ Penicillin allergy is reported in 10% to 20% of patients in clinical practice^2^, ^3^, ^4^ but it has been shown that most of these patients do not have true allergies and are able to tolerate penicillins after thorough allergy evaluation.^5^ Patients who are labeled penicillin‐allergic may be prescribed less effective, more expensive or more toxic drugs, leading to increased healthcare costs, and antimicrobial‐resistant infections.^6^ Certain populations such as patients with malignancies, human immunodeficiency virus (HIV) infection and recurrent sinusitis or urinary tract infections are more likely to require multiple courses of antibiotics and benefit from appropriate allergy labeling.^7^

The importance of penicillin allergy delabeling has been recognized by antibiotic stewardship programs.^8^, ^9^ However allergy testing is time‐consuming and has inherent risks as even skin tests may trigger anaphylaxis.^10^ Furthermore, despite efforts to remove allergy labels, patients who test negative may not subsequently receive the antibiotics tested due to accidental relabeling^11^ or patients' and physicians' perceptions^12^ which may result in persistence of incorrect allergy labels.

In this study, our primary aim was to determine the clinical value and safety of β‐lactam allergy evaluation performed in a dermatology outpatient clinic. Our secondary aims were to evaluate patient usage of antibiotics following negative testing and identify factors for nonusage.

## 2 METHODS

We performed a 7‐year, retrospective medical record review of patients over the age of 16 years old who underwent skin tests and/or drug provocation tests (DPT) to any β‐lactam antibiotics at the drug eruption clinic in National Skin Centre, Singapore from 1st January 2010 to 31st December 2016. This study was performed as an audit on safety and quality of patient care.

Careful history taking and examination were performed in all patients. We corroborated patients' drug histories with electronic medical records when available and by contacting physicians involved in the care of the patients when necessary. We determined the types of cutaneous and systemic reactions, time to onset of reaction after drug consumption and comorbidities. Drug hypersensitivity reactions were considered “immediate” if onset of symptoms occurred within 1 to 6 hours of last dose of drug, and “delayed” if occurred after 6 hours. Patients with reactions strongly suggestive of anaphylaxis were not evaluated further in our clinic due to safety reasons and centre's policy. These cases were referred to a general hospital with emergency or intensive care facilities.

In patients with history suggestive of delayed reactions, patch tests (PTs) with crushed commercial tablets in 30% white soft paraffin were performed as per guidelines^13^ and readings done at day 2 and 4 according to International Contact Dermatitis Research Group criteria. In patients with initial reaction of uncertain nature or suggestive of immediate hypersensitivity, skin prick tests (SPT) followed by intradermal tests (IDT) were performed in accordance with previous recommendations^14^ with penicillin G, ampicillin, amoxicillin‐clavulanic acid and DAP Penicillin Test Kit (Diater; Madrid, Spain) which consisted of benzylpenicilloyl poly‐l‐lysine (PPL) and minor determinant mixture (MDM). PPL and MDM were replaced on 1 December 2011 by benzylpenicilloyl octa‐l‐lysine and the minor determinant (sodium benzylpenilloate), respectively. SPT/IDT with delayed reading at 24 to 48 hours were performed in some patients with uncertain reaction or delayed type reaction. In patients labeled allergic to a cephalosporin, SPT/IDT to the above and the labeled cephalosporin (if available in intravenous form) was also done. If the cephalosporin did not exist in intravenous form (eg, cephalexin), only SPT to the pulverized commercial tablet was performed.

Direct DPTs were performed in some cases if patients declined skin tests and their reactions were considered to be of low risk (ie, without signs of angioedema, anaphylaxis, pustulosis, mucositis, blisters, erosions, or painful skin lesions). Completion of DPT was necessary to conclude drug allergy evaluation.

DPTs to the labeled β‐lactam were performed in general accordance with guidelines of the European Network for Drug Allergy.^15^ DPT was performed without blinding, either as a single therapeutic dose challenge or graded challenge given as one‐quarter, one‐half followed by full single therapeutic dose with 60 to 75 minutes intervals between doses. These dose escalation protocols, which differ from guidelines, were used as we only evaluated patients with low‐risk reactions. Smaller starting doses such as 1% and 10% which are more appropriate for patients with anaphylaxis were hence not employed. Patients were observed in clinic for 120 to 150 minutes after the final dose. Extended DPT with normal therapeutic doses was performed if the drug was strongly suspected in the initial delayed‐type reaction. The duration of extended challenge was not standardized and could be up to the number of days from initiation of the antibiotic to the index reaction. We considered DPT to be positive only if objective signs were elicited within a reasonable time frame. SPT/IDT positive patients did not proceed to DPT but given an option to evaluate for selective β‐lactam hypersensitivity if SPT/IDT was positive to amino‐penicillins.

**Figure 1:**
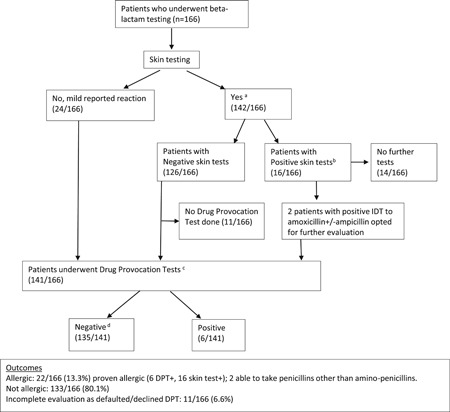
Flowchart of patients undergoing skin tests and oral provocation tests and outcomes. ^a^One hundred twenty SPT/IDT, 15 SPT/IDT with delayed reading, eight PT, five SPT/IDT, and PT. ^b^Six IDT amoxicillin‐clavulanic acid only + (including one on delayed reading); four IDT amoxicillin‐clavulanic acid+  and ampicillin+ (including one on delayed reading); one IDT amoxicillin‐clavulanic acid+  and penicillin G+; one IDT amoxicillin‐clavulanic acid+ , penicillin G + , ampicillin+ ; two IDT ampicillin+ (including one on delayed reading); one IDT penicillin G+; one IDT penicillin G+ and MDM+. ^c^One hundred thirty seven graded challenges, five single dose challenges; 19 had extended DPTs (two up to 7 days duration). ^d^Includes the two patients with positive IDT ampicillin (DPT penicillin V −ve). Number of tests exceeds number of patients as some patients had tests to multiple β‐lactams. IDT, intradermal test; MDM, minor determinant mixture; SPT, skin prick test

Conclusion of drug allergy evaluation included counseling of patients regarding antibiotic tolerance, modification of antibiotic allergy labels in patients' records in the nation‐wide electronic allergy notification system and a letter to patients' managing physicians about their changed allergy status. Figure 1 shows how the patients in our study were evaluated.

We performed a follow‐up evaluation of β‐lactam antibiotic use in patients who were proven nonallergic. This was performed by telephone call and/or through electronic medical records if available. If β‐lactams had been used, we attempted to determine if any adverse reaction occurred. If β‐lactams had not been used, we asked for reasons for nonuse, for example, patients' or physicians' concerns or absence of indication.

## 3 RESULTS

### 3.1 Baseline demographics

Table 1 summarizes the characteristics of the 166 patients included. The median age of our patients was 42 (range, 14‐76) years. There were 96 females (57.8%) and 70 males (42.2%). The ethnic distribution was as follows: 141 Chinese (84.9%), 10 Malay (6.0%), 5 Indian (3.0%), 4 Caucasian (2.4%), 2 Eurasian (1.2%) patients, and 4 (2.4%) others.

**Table 1:** Baseline demographics of patients included in the study

	No (%) of patients, *N* = 166
Sex	
Female	96 (57.8)
Male	70 (42.2)
Median age, range, y	42 (14‐76)
Recurrent infections or predisposing conditions^a^	44 (26.5)
Multiple antibiotic allergy labels	21 (12.7)
More than one β‐lactam^b^	6 (3.6)
Antibiotic class(es) other than β‐lactams	15 (9.0)
One	12 (7.2)
Two	2 (1.2)
Three	1 (0.6)
β‐Lactam allergy labels	173
Penicillins	158 (91.3)
Penicillin	81 (46.8)
Amoxicillin/amoxicillin‐clavulanic acid	75 (43.4)
Cloxacillin	2 (1.2)
Cephalosporins	13 (7.5)
Cephalexin	11 (6.4)
Ceftriaxone	1 (0.6)
Cefuroxime	1 (0.6)
Aztreonam	1 (0.6)
Meropenem	1 (0.6)
Type of reaction, based on time to onset	173
Immediate, <1 up to 6 h	38 (22.0)
Delayed, >6 h	52 (30.1)
Uncertain	83 (48.0)
Nature of rash^c^	173
Unknown/nonspecific	78 (45.1)
Urticaria only	22 (12.7)
Urticaria + angioedema	46 (26.6)
Maculopapular exanthema	27 (15.6)

^a^ Includes recurrent urinary tract infection, recurrent sinusitis, diabetes mellitus, bronchiectasis, eczema, valvular heart disease, HIV infection, and malignancy.

^b^ Includes three cases with penicillin and cephalexin, two cases with cephalexin and amoxicillin‐clavulanic acid, one case with penicillin, ceftriaxone and meropenem labels.

^c^ Includes cases of anaphylaxis are not evaluated in our clinic.

There were 44 patients (26.5%) who had a history of recurrent infections (eg, urinary tract infections or sinusitis) or conditions which predisposed them to future infection (eg, bronchiectasis, diabetes mellitus, HIV infection, malignancies, valvular heart disease, or eczema).

### 3.2 Allergy labels

The 166 patients in our study had 173 β‐lactam allergy labels. These were predominantly of the penicillin group (158 labels, 91.3%) and included 81 (46.8%) penicillin, 75 (43.4%) amoxicillin, or amoxicillin‐clavulanic acid and 2 (1.2%) cloxacillin labels. There were 11 (6.4%) cephalexin allergy labels and 1 (0.6%) label each to ceftriaxone, cefuroxime, aztreonam, and meropenem. A total of 21 (12.7%) patients had multiple antibiotic allergy labels, including 15 (9.0%) who also had labels to non‐β‐lactam antibiotics. A total of 6 (3.6%) patients had allergy labels to two or more β‐lactams.

### 3.3 Reported allergic reactions

Out of the 173 allergy labels, there were 38 (22.0%) reported immediate‐type reactions, 52 (30.1%) delayed‐type reactions, and 83 (48.0%) were classified as uncertain as details were not available or not recalled. The following types of skin lesions were reported: 22 (12.7%) urticaria only, 46 (26.6%) urticaria with angioedema, 27 (15.6%) maculopapular exanthema, and 78 (45.1%) rashes of unknown or uncertain nature.

### 3.4 Results of allergy evaluation

The time lapse between reported reaction and allergy label evaluation was as follows: less than 6months (57 labels, 32.9%), 6 months to 1 year (12, 6.9%), 1 to 3 years (8, 4.6%), greater than 3 years (93, 53.8%), and unknown (3, 1.7%). One hundred and forty two patients underwent skin tests for their 148 β‐lactam allergy labels. Of these 142 patients, 120 (81.1%) underwent SPT/IDT, 15 (10.1%) underwent SPT/IDT with delayed reading, eight (5.4%) underwent patch testing, and five (3.4%) underwent both patch testing and SPT/IDT with delayed reading. There were 16 positive skin tests: six IDT amoxicillin‐clavulanic acid+ (including one on delayed reading); four IDT amoxicillin‐clavulanic acid+ and ampicillin+ (including one on delayed reading); one IDT amoxicillin‐clavulanic acid+ and penicillin G+; one IDT amoxicillin‐clavulanic acid+, penicillin G+ and ampicillin+; two IDT ampicillin+ (including one on delayed reading); one IDT penicillin G+; one IDT penicillin G+ and MDM+. Thirteen immediate type and three delayed‐type hypersensitivity were diagnosed on SPT/IDT. None of our PTs were positive.

Eleven patients who had negative skin tests did not return for DPT and evaluations were considered inconclusive. Out of the 115 patients with negative skin tests who proceeded to DPT, 110 patients tested negative. Negative predictive value for skin testing was 95.6%.

There were 141 patients who underwent 142 DPT. This group comprised of 24 patients who proceeded to 25 DPT directly, including one patient who underwent two DPTs, 115 patients who had negative skin tests and two patients with positive IDT on delayed reading to aminopenicillins who tolerated DPT to penicillin V. Five (3.5%) DPT were performed as a single therapeutic dose challenge while 137 (96.5%) were graded challenges. Details of six patients who had positive DPT are shown in Table 2. Nineteen patients (13.4%) underwent extended DPT lasting 2 to 7 days without reaction.

**Table 2:** Details of positive drug provocation tests

	Age/sex	Drug	Type of reaction based on time to onset	Rash reported	Skin test/drug provocation test	Time lapse since last exposure to tests	Type of reaction (rash)	Time from first dose DPT	Treatment
1	22/F	Penicillin	Unclear	Urticaria	SPT/IDT penicillins	<12 mo	Delayed (MPE)	>6 h	Topical steroids
					DPT amoxicillin (graded, separate day from SPT/IDT)				
2	36/F	Amoxicillin‐clavulanic acid	Delayed	Unknown (lip swelling)	SPT/IDT penicillins	<6 mo	Delayed (FDE)	4 h	Topical steroids
					DPT amoxicillin‐clavulanic acid (graded)				
3	23/M	Cephalexin, penicillin	Immediate (almost 6 h to onset)	Urticaria + angioedema	SPT/IDT penicillins	<6 mo	Immediate (generalized urticaria; no angioedema)	2.5 h	IM diphenhydramine
					SPT cephalexin				PO prednisolone; observation in emergency department
					DPT cephalexin (graded)				
4	44 /F	Amoxicillin	Unknown	Unknown (possibleurticaria)	Declined skin tests	>3 y	Immediate (mild urticaria)	2 h	Antihistamines
					DPT amoxicillin				
5	29/F	Cephalexin	Unclear	Unknown (eczema vs MPE due to drug vs infection)	PT cephalexin	6 wk	Immediate (generalized urticaria; no angioedema)	5 h	Antihistamines
					DPT cephalexin (graded)				
6	49/F	Amoxicillin	Unknown	Unknown	SPT/IDT penicillins	>3 y	Delayed (MPE)	48 h	Treated elsewhere
					DPT amoxicillin (graded, same day as SPT/IDT)				

*Note*: SPT/IDT penicillins, skin prick test/intradermal test to penicillin G, benzylpenicilloyl‐poly‐l‐lysine (replaced by benzylpenicillolyl octa‐l lysine after 2013), minor determinant mixture (replaced by minor determinant after 2013).

Abbreviations: ampicillin, amoxicillin‐clavulanic acid; DPT, drug provocation test; FDE, fixed drug eruption; PT, patch test; MPE, maculopapular exanthem.

In summary, we proved that 133 patients were not allergic to β‐lactams, which constituted 85.8% of the 155 patients who were conclusively evaluated. Twenty‐two (13.3%) patients had confirmed allergy to β‐lactams −16 had immediate hypersensitivity (13 SPT/IDT+, three DPT+) and six had delayed hypersensitivity (three SPT/IDT with delayed reading+, three DPT+). Two patients with delayed hypersensitivity were tolerant of β‐lactams other than amino‐penicillins. Eleven (6.6%) patients did not complete their evaluation. Negative predictive value of skin testing was 95.9%. Reactivity of skin tests did not seem to be related to time lapsed since initial reaction. Out of 16 positive skin tests, 10 (seven immediate reactions and three IDT with delayed reading) were performed within 12 months of initial reaction, while six (all immediate reactions) after 3 years had passed.

### 3.5 Safety

None of the reactions were life‐threatening nor severe requiring admission or subcutaneous adrenaline.

Three positive reactions to DPT occurred after or at the end of the period of clinic observation. One patient who reported mild urticaria as initial reaction declined skin tests and underwent amoxicillin DPT directly. He developed mild urticaria within 2 hours of starting DPT at a cumulative dose of 750 mg amoxicillin. Rescue treatment with cetirizine 10 mg was given with good response.

Two patients labeled allergic to cephalexin, who had negative SPT to cephalexin and negative SPT/IDT to penicillins, developed generalized urticaria within 2.5 and 5 hours respectively after starting graded DPT. The first patient reacted after a cumulative dose of 375 mg, while the second patient reacted after a cumulative dose of 875 mg. Both patients remained hemodynamically stable. One patient required intramuscular diphenhydramine and systemic steroids and further observation in the emergency department.

One patient who reported a vague initial reaction with possible lip swelling after taking amoxicillin‐clavulanic acid underwent evaluation as for immediate reaction with SPT/IDT and subsequent DPT, but was eventually diagnosed with amoxicillin‐clavulanic acid‐induced FDE after reproduction of the rash with DPT.

### 3.6 β‐Lactam use after allergy evaluation

Out of 135 patients who had β‐lactam allergy label modification, 45 (33.3%) were not contactable and had no record of re‐exposure according to available records in public medical institutions. Thirty (22.2%) patients had taken a β‐lactam antibiotic in the posttesting period, with only one developing a reportedly “mild” reaction. Fifty‐two patients had not used a β‐lactam. Thirty‐five (25.9%) patients had no indication, while 17 (12.6%) avoided β‐lactam due to concerns of allergy (two on the part of the attending physician, 12 of the patient, and three of both the patient and physician).

## 4 DISCUSSION

Inappropriate allergy labels result in increased healthcare costs and adverse events.^16^, ^17^ Our results show that more than 85% of penicillin allergy labels in our patient group were incorrect. This finding is consistent with those of other reports^2^, ^5^ and supports the need for allergy evaluation services in our healthcare system.

Skin testing in our population had high NPV of 95.9% which is consistent with reported literature^18^, ^19^ and reassuring for patients who are afraid to proceed to direct DPT. Fourteen (87.5%) out of 16 positive skin reactions were to aminopenicillins. MDM testing was positive in only one patient, who also had a positive reaction to penicillin G. None of our patients tested positive to PPL. These results may demonstrate the decreasing importance of PPL and MDM testing, reflecting the dominant use of aminopenicillins and cephalosporins in current clinical practice. Bourke et al similarly reported that the use of PPL and MDM did not improve NPV in skin testing^20^ and further studies are needed to evaluate this. In our cohort, these findings may be due to patient selection as the usefulness of minor determinant testing has been shown in more severe anaphylaxis cases.^21^ Twelve positive skin test reactions were to aminopenicillins only but testing of tolerance of other β‐lactams was done in only two of our patients. Improved understanding of side‐chain allergy should reassure patients and allergists in further evaluation of β‐lactams with different side chains if required.

Skin testing for cephalosporin allergy is not as well‐standardized as for penicillins and may pose specific difficulties. In our two cephalexin‐allergic patients, the lack of an intravenous form of cephalexin for IDT and the direct course to DPT after SPT resulted in the most severe reaction in our study population. Skin testing with amino‐penicillins with similar side chains did not seem to be useful in our evaluation of cephalexin allergy. We recommend strict adherence to guidelines recommending lower starting doses of 10% (1%, if required) of full therapeutic dose in such situations.^15^, ^22^

We observed that positive reactions to DPT may occur several hours into the test procedure. This demonstrates the need for long observation periods which may also be reassuring for patients. Clear instructions need to be given to patients after completion of DPT doses and allergist should verify nonreaction several days after DPT before removal of allergy labels.

We experienced no safety issues in our population as we excluded high‐risk cases. However, the lack of reliable history of the initial reaction remains a problem as seen from four out of six of positive DPTs. FDE, in particular, may be mistaken for angioedema if perioral area is involved and post‐inflammatory hyperpigmentation is not obvious, resulting in the wrong choice of skin test for evaluation as skin testing for FDE is only reactive in lesional skin. The limited clinical utility of patients' history in predicting skin test reactivity has been previously reported.^23^, ^24^

Eleven patients who underwent skin tests did not complete their evaluation by following up with DPT. This is a wasteful consumption of resources and patients need to be counseled that while skin testing has its value, DPT remains the conclusive step. It has been suggested that direct DPT should be considered in carefully selected patients in appropriate clinical settings.^25^, ^26^, ^27^

Many patients did not take β‐lactams despite negative tests often due to continued perceived intolerance. This lowers the effectiveness of allergy label modification which has also been reported in other studies.^11^, ^20^ There is a need for patient and clinician education as well as systemic measures (eg, electronic allergy record modification) to facilitate β‐lactam use in proven‐tolerant patients.^25^ Improvements in electronic allergy records systems should also keep pace with developments in understanding of side‐chain allergy and allow documentation of tolerated β‐lactams.

Our study is limited in some ways. We did not specifically collect data for in vitro tests in this study but we did not have positive results for IgE to penicilloyl G, penicilloly V, amoxicillin, or ampicillin during this period of time. The retrospective nature of the study may result in incomplete data but all patients were reviewed and had careful documentation performed by dermatologists (YKH and YLL) with expertise in drug hypersensitivity testing. Our follow‐up survey of β‐lactam use after testing was limited as not all patients were contactable but we were nevertheless able to recognize that patients and physicians feared re‐exposure to β‐lactams despite negative testing, a finding similar to other studies.^11^, ^20^ Our small sample size and retrospective study design limited the analysis of factors which may have predicted positive test reactions.

## 5 CONCLUSION

We confirmed that β‐lactam allergy evaluation and delabeling is safe in carefully selected cases and the proportion of patients who have true allergic reaction is low. There remains a need for faster diagnostic methods to evaluate β‐lactam allergy. Skin tests are of value in cases with moderate risk or of uncertain reaction but direct DPT may be considered in low‐risk cases. Starting doses in DPT should be low to minimize severe reactions, especially in unknown or severe reactions. Allergy label removal must be accompanied by patient education and adequate documentation to facilitate future use. More studies are needed to understand factors from patients' and physicians' perspectives which may impede allergy delabeling efforts.

## CONFLICT OF INTERESTS

The authors declare that there are no conflict of interests.

## ETHICS STATEMENT

This study was performed as an audit of clinical practices approved by National Skin Centre, Singapore.

## Data Availability

The data that support the findings of this study are available from the corresponding author upon reasonable request.
